# A Social Ecological Approach to Addressing Childhood Trauma

**DOI:** 10.1002/puh2.70091

**Published:** 2025-08-12

**Authors:** Sarah Pember, Michele L. Pettit

**Affiliations:** ^1^ Department of Public Health & Community Health Education University of Wisconsin‐La Crosse La Crosse Wisconsin USA

**Keywords:** childhood trauma, positive childhood experiences, social ecological model

## Abstract

Exposure to trauma has a detrimental impact on the social, emotional, and cognitive development of youth with lasting impacts into adulthood and, thus, represents a leading concern for all public health professionals, as the impacts are seen most immediately in schools but require attention at all levels of a community. This article presents strategies and interventions for addressing childhood trauma at each level of the Social Ecological Model. Schools represent an ideal setting for addressing childhood trauma through mindfulness‐based practices, trauma‐related training for teachers, and resources and support for parents and caregivers. However, such initiatives should be complemented by community‐level strategies and interventions for preventing and addressing trauma as well as policies supportive of children and families. Childhood trauma is a complex issue that requires intervention at the intrapersonal, interpersonal, institutional/organizational, community, and policy levels. School and community health professionals can play a vital role in intervening at each of these levels and promoting positive childhood experiences (PCEs) to achieve the best possible outcomes for youth and their families.

## 1 Introduction

For more than two decades, childhood trauma increasingly has gained traction among school and community health professionals. The Adverse Childhood Experience (ACE) study, a landmark investigation co‐led by the Centers for Disease Control and Prevention (CDC) and Kaiser Permanente from 1995 to 1997, brought national attention to the impact of “childhood exposure to abuse and household dysfunction” on morbidity and mortality among Americans [1, 2, p. 248].

Trauma can manifest in many forms, such as exposure to violence, discord, substance use, or mental health concerns in the home. Such exposure can result in various detrimental health, educational, and occupational outcomes across the lifespan [3] and can impact how individuals perceive themselves and the world around them [4]. As such, trauma is perhaps one of the most important public health issues facing youth today.

According to the Substance Abuse and Mental Health Services Administration (SAMHSA) [4], “Individual trauma results from an event, series of events, or set of circumstances that is experienced by an individual as physically or emotionally harmful or life‐threatening and that has lasting adverse effects on the individual's functioning and mental, physical, social, emotional, or spiritual well‐being.” (p. 7). Inherent to this definition of trauma are the three “E's”—the event, the experience of the event, and the effect(s) of the event [4].

According to the literature, exposure to ACEs is widespread. For example, in the original ACE study, Felitti et al. [2] reported that 52% of their sample of US adults had undergone at least one traumatic event and 6.2% had undergone ≥4 traumatic events. In a recent study, Swedo et al. [5] analyzed data from the Behavioral Risk Factor Surveillance System (BRFSS) from 2011 to 2020 and found that “63.9% of US adults reported at least one ACE” (e.g., having a parent/caregiver with a mental illness or substance use disorder and experiencing abuse or neglect) and “17.3% reported four or more ACEs” (p. 707).

On a global scale, the rates are even higher. In fact, Benjet et al. [6] sought to investigate the global burden of trauma by examining results from the World Health Organization's World Mental Health Surveys. Specifically, they reported the prevalence of trauma among adults across the income spectrum from 24 countries and found that more than 70% had experienced a traumatic event. Moreover, 30.5% had experienced ≥4 traumatic events.

In the past 5 years, the term “trauma‐informed” has emerged as a buzzword, and educational and health care organizations, as well as communities across the country, increasingly have initiated efforts to become “trauma‐informed.” The impetus behind these efforts is a renewed commitment to improved health and academic outcomes for youth adversely affected by trauma [7, 8]. Although there are many ways trauma‐informed practices are defined [9, 10], the SAMHSA [4] defines the term “trauma‐informed” as follows:

A program, organization, or system that is trauma‐informed realizes the widespread impact of trauma and understands potential paths for recovery; recognizes the signs and symptoms of trauma in clients, families, staff, and others involved with the system; and responds by fully integrating knowledge about trauma into policies, procedures, and practices, and seeks to actively resist re‐traumatization. (p. 9)

Not only do school and community health professionals need to be trauma‐informed to alleviate the impact of ACEs, but they also need to promote positive childhood experiences (PCEs). PCEs can be broadly categorized as nurturing relationships, promoting stable safe environments, providing opportunities to develop connections, and strengthening emotional competencies [11]. PCEs include the support of family and friends, a sense of belonging at school and in the community, feeling safe and protected at home, and having non‐parent adult mentors such as school health professionals who genuinely care. PCEs show a dose–response relationship in preventing adult depression and poor mental health, acting as a buffer against trauma and leading to more positive social and emotional outcomes into adulthood [12]. The Social Ecological Model provides an appropriate framework for promoting trauma‐informed practices and PCEs in schools because it “focuses attention on both individual and social environmental factors as targets for health promotion interventions” [13, p. 351].

Much of the work in childhood trauma exclusively has focused on either school‐ or community‐based intervention and support and working with youth or their families directly. What is needed is purposeful attention to not only the individual and organizational efforts that can be initiated to address childhood trauma but also to the systems and structures that can allow that work to be successful, which is why a social ecological approach is needed.

## 2 The Social Ecological Model

According to McLeroy et al. [13], the Social Ecological Model involves five levels of intervention, including intrapersonal (e.g., individual factors such as knowledge, attitudes, and behaviors), interpersonal (e.g., entities outside of the individual such as family members, friends, peers, and neighbors), institutional/organizational (e.g., entities such as schools, workplaces, and places of worship), community (e.g., neighborhoods and the broader community), and public policy (e.g., local, state, and federal laws). The purpose of this article is to provide strategies for school and community health professionals, as well as policy makers, to address childhood trauma at each level of the Social Ecological Model.

## 3 Reflexivity Statement

The authors have spent multiple years working with students in K‐12 and college settings who experienced varying degrees of trauma and ACEs. Additionally, they have taught future educators and public health professionals about the need to consider trauma and the social determinants of health in their approach to working with individuals and communities. Their personal and professional experiences inform their view on the importance of addressing childhood trauma at all levels of the Social Ecological Model.

## 4 Discussion

### 4.1 Implications for Public Health Research

In its 2022 report, *Adverse Childhood Experiences Research Priorities for Equitable Prevention, Intervention, Identification, and Response*, the CDC outlined gaps and priorities regarding ACEs in the scientific literature. Examples of areas for future research include investigating traumatic experiences not reflected in the original ACEs study, measuring ACEs in a way that addresses the impact of the social determinants of health (e.g., racism and poverty), further examining risk and protective factors related to ACEs, and determining evidence‐based practices for preventing and responding to ACEs [14].

### 4.2 Implications for Public Health Policy, Practice, and Equity

In 2021, US Surgeon General Vivek Murthy issued an advisory on youth mental health, thus sounding the alarm on a crisis in the making [15]. Two years later, Dr. Murthy released another advisory on the dangers of social media in relation to youth mental health [16]. Not only did the Surgeon General call attention to the mental health needs of today's youth, but the American Academy of Pediatrics, American Academy of Child and Adolescent Psychiatry, and Children's Hospital Association declared a “national emergency in child and adolescent mental health” in 2021 [17]. Moreover, professional organizations like the American Psychological Association have led advocacy efforts to support youth access to mental health services [18]. Despite such advocacy efforts, schools continue to face funding deficits and shortages of mental health professionals [19]. To that end, the need for multi‐level strategies and interventions for coping with childhood trauma has never been greater.

#### 4.2.1 Intrapersonal Level

A plethora of studies have demonstrated the positive impact of mindfulness‐based practices (e.g., “present moment awareness/techniques,” breathing exercises, meditation, body scans, and yoga) on youth mental health. For example, Moyes et al. [20] examined multiple studies and uncovered evidence supportive of mindfulness‐based interventions for youth and adults affected by ACEs. Moreover, Borquist‐Conlon et al. [21] conducted a meta‐analysis to examine the efficacy of mindfulness‐based interventions (e.g., “present moment awareness/techniques,” breathing exercises, meditation, body scans, and yoga) on youth anxiety. Results from their analysis revealed that mindfulness‐based practices were effective in helping youth manage anxiety. Similarly, Coholic et al. [22] investigated numerous studies to explore the usefulness of mindfulness‐based practices that incorporate creative expression (e.g., drawing, sculpting, music, and poetry) among youth. Results from their research indicated that arts‐based mindfulness practices especially were helpful for youth undergoing difficult challenges.

A final example of the power of teaching youth mindfulness‐based skills that they can apply to their individual lives is the Kindness Curriculum developed by researchers at the Center for Healthy Minds. The Kindness Curriculum focuses on “the senses, breath, and body movements” that are essential to cultivating mindfulness as well as the practice of expressing kindness and compassion toward oneself and others. The Kindness Curriculum has been associated with positive “academic performance, peer relationships, and teacher‐perceived social competence” and is an example of the ripple effect that can come from teaching youth skills for practicing mindfulness and kindness [23]. Such skills are especially beneficial for LGBTQ+ youth and other marginalized groups who are vulnerable to trauma associated with identity‐based bullying [24].

#### 4.2.2 Interpersonal Level

Teaching youth mindfulness‐based practices requires training for teachers who facilitate the classroom practices. Moreover, such practices may not be suitable for all students impacted by trauma. To that end, such practices can be complemented by story circles. Rooted in Indigenous cultures, story circles effectively have been used to process traumatic experiences in safe spaces like faith‐based settings and clinical environments. More recently, they have been used by teachers in educational settings to facilitate conversation about youths’ experiences with trauma. Specifically, youth regularly meet with the same peers and are led by a teacher who helps them establish ground rules for sharing, asks questions, and offers discussion prompts. These consistent opportunities for sharing stories in a calm, structured, and affirming environment contribute to a “caring learning community” in which youth feel comfortable and supported [25].

#### 4.2.3 Institutional/Organizational Level

At the institutional/organizational level, trauma‐related training for teachers is imperative. The National Child Traumatic Stress Network (NCTSN) developed a “Child Trauma Toolkit for Educators.” The toolkit offers educators information and resources for supporting youth affected by trauma in preK‐12 settings [26]. The Mental Health First Aid training is another option for preparing educators with the skills necessary to “identify, understand, and respond” to the mental health needs of youth [27].

Not only is there a need for school and community health professionals to be trained on how to respond to trauma, but there also is a need for these individuals to develop compassion and resilience. According to WISE: Initiative for Stigma Elimination, a national organization created to decrease stigma related to mental illness and addiction, compassion resilience refers to “the ability to maintain emotional, mental, and physical well‐being while compassionately supporting others through the challenges of daily work. It involves maintaining empathy, strength, and hope in the face of adversity while taking steps to prevent compassion fatigue.” To that end, WISE created toolkits for educators, health and human service providers, and parents/caregivers to help them build resilience—a key protective factor in offsetting the impact of trauma and compassion fatigue [28].

#### 4.2.4 Community Level

Trauma impacts individuals, but its effects are compounded by the nature of the communities in which individuals live. The risks for childhood trauma and the long‐term negative health and opportunity outcomes are compounded by existing inequities [14], resulting in higher proportions of children of color and those in lower income families enduring traumatic experiences [29].

The Center for Community Resilience at the Milken Institute School of Public Health is a leader in promoting methods and models for addressing childhood trauma through advancing equity and resilience at the community level. Ellis and Dietz [30], lead researchers at the institute, emphasize a “Pair of ACEs” as the interconnection between ACEs and Adverse Community Environments—environments with persistent discrimination, poverty, lack of opportunity, poor housing, and violence—and recognize that addressing childhood trauma requires addressing systemic issues. Trauma‐informed practices must be applied at the community level. Their building community resilience (BCR) model demonstrates the importance of multiple stakeholders working together to prevent not only individual traumas but also to address the disparate conditions in which children live and learn [30, 31].

Schools represent a key stakeholder for providing trauma‐related education, resources, and support to parents, caregivers, and families of youth and demonstrate how changes and interventions at an institutional/organizational level can benefit both the community and the interpersonal relationships within it. Because trauma often is intergenerational, parents of children with ACEs can benefit from the support provided by social workers, school counselors, pediatricians/family practitioners, and so forth. Such providers can be essential in assisting parents with navigating mental health crises and the mental health care system [32].

The Resilience and Trauma‐Informed Community framework, a regional framework used to address trauma across the state of Wisconsin, emphasizes a “kaleidoscope of change” that, similar to the BCR model, is grounded in a shared understanding among community partners about the nature and effects of trauma. The community partners work together to reduce the risks of trauma through prevention and intervention, strengthen resilience by enhancing protective factors throughout the community, and ensure that there is support for healing and recovery [33]. Community‐level strategies support individuals by explicitly addressing systemic racism and other forms of discrimination. Although ground‐up efforts among and across communities themselves are important, top‐down strategies and policy reforms are necessary to address the needs of children and families and therefore to both prevent and address the occurrence and experience of trauma.

#### 4.2.5 Policy Level

It is important to emphasize school‐based practices that are trauma‐sensitive and focus the work of administrators, teachers, and school‐based health officials on addressing the immediate needs of children and families. However, it should not be up to schools and institutions/organizations alone to address these needs. For that reason, policy change is necessary to create environments in which the positive impacts of trauma‐sensitive practices can be maintained.

The CDC's Essentials for Childhood Framework emphasizes the need to create safe, stable environments for children, with caregivers who can form and sustain nurturing, protective relationships with those children, as key for addressing and eliminating childhood traumatic experiences. Addressing childhood trauma means addressing the factors that prevent caregivers from being able to do just that. One major factor is economic stress. Populations experiencing economic stress have been identified by the NCTSN [34] as being at greater risk for childhood trauma. As caregivers attempt to cope with financial insecurity, the children suffer as well. It also is evident in the data that lower SES children are at greater risk for experiencing ACEs. Both the CDC [35] and the Center for Community Resilience [36] have identified stronger economic supports for families and access to high‐quality, affordable early childhood education and childcare as key policy initiatives for addressing childhood trauma. The rising cost of childcare combined with declining options for parents has created an “untenable” situation in this country [37]. Universal early childhood education and State Dependent Care Tax Credits (SDCTC) could help address the gaps in care and disparities in education. A national expansion of the Child Tax Credit could lift as many as 400,000 children out of poverty in its first year of implementation [38]. The effects of expanding the Child Tax Credit were witnessed in the dramatic jump of childhood poverty from a low of 5.2% in 2021 to 12.4% in 2022, as pandemic‐era supports expired [39].

Another economic support that may reduce toxic stress and suffering that can result in child maltreatment is paid family leave, which has been shown to reduce familial violence [40]. Paid parental leave also is associated with lower levels of postpartum depression and increased parental engagement [41]. No permanent national legislation has ever been passed, and the 2022 KFF Women's Health Survey found that only 43% of women report receiving some form of parental leave from their employer [42].

Ensuring access to affordable, effective mental health care and substance use treatment also would help to alleviate the traumatic impact of living with a parent who is struggling with mental health concerns and/or substance abuse. The latest Survey of Mental Health in America [43] reports that of the 15.35% of US adults with a substance use disorder, 93.5% did not receive treatment. Additionally, nearly one‐third (28.2%) of adults experiencing any form of mental illness did not receive the treatment they needed; of those, 42% could not do so because it was cost prohibitive [43]. Although these statistics do not explicitly address how many caregivers are living with these illnesses, legislation to provide for sufficient mental health care could help change the trajectory of mental illness for those who are and, ultimately, support a more stable environment for the children these adults may care for.

Addressing systemic racism and other forms of discrimination that perpetuate disparities in social determinants of health across communities, families, and, ultimately, children, can not only be accomplished through economic supports but also through policies that provide equitable access to information, resources, and services. For example, policies that increase access to voting and health care could assist in dismantling structural racism [44]. Ultimately, the end goal of any policy‐level change is to ensure that children are living in safe, stable home environments, where they are supported because their caregivers are as well.

## 5 Conclusions

Addressing the vast and troubling prevalence of childhood trauma requires an ecological approach, with efforts made in both prevention and intervention and emphasis on PCEs. These efforts are essential at the individual level to support children as they grow and avoid or overcome trauma, but also to effect the changes needed so that families, schools, and communities can provide the safe, stable environments required for children to thrive. An effective approach to addressing childhood trauma and its lasting impacts will require not only school‐based health officials but also community practitioners and policy makers as well. Such an approach should start with the development and implementation of policies to support the capacity of caregivers to provide PCEs and continue by working with families directly through school‐based initiatives or other community‐based interventions to help guide or create opportunities for PCEs. By addressing childhood trauma at every level of the Social Ecological Model, every child can have the connections and support they need to either avoid or overcome adversity—not just go through it, but grow through it.

## Author Contributions

**Sarah Pember**: writing – original draft, writing – review and editing, conceptualization, resources. **Michele L. Pettit**: writing – original draft, writing – review and editing, resources, conceptualization.

## Ethics Statement

This article adheres to the ethical policies of the journal.

## Conflicts of Interest

The authors declare no conflicts of interest.
